# Clinical–Pathological Features of Thyroid Neoplasms in Young Patients Diagnosed in a Single Center

**DOI:** 10.3390/life14060696

**Published:** 2024-05-28

**Authors:** Aura Jurescu, Dan Brebu, Alexandra Corina Faur, Octavia Vita, Robert Barna, Adrian Vaduva, Oana Popa, Anca Muresan, Mihaela Iacob, Marioara Cornianu, Remus Cornea

**Affiliations:** 1 Department of Microscopic Morphology-Morphopatology, ANAPATMOL Research Center, “Victor Babes” University of Medicine and Pharmacy, 300041 Timisoara, Romania; 2 Researching Future Chirurgie 2, Department of Surgery II, “Victor Babes” University of Medicine and Pharmacy, 300041 Timisoara, Romania; 3 Department of Anatomy and Embriology, “Victor Babes” University of Medicine and Pharmacy, 300041 Timisoara, Romania; 4 Department of Pathology, “Pius Brinzeu” County Clinical Emergency Hospital, 300723 Timisoara, Romania; 5 Department of Endocrinology, Centre of Molecular Research in Nephrology and Vascular Disease, “Victor Babes” University of Medicine and Pharmacy, 300041 Timisoara, Romania

**Keywords:** thyroid cancer, young patients, papillary carcinoma, risk factor, lympho-vascular invasion, extrathyroidal extension, locoregional lymph node invasion

## Abstract

*Background and objectives:* The aim of this study was to evaluate the clinical–pathological profile in young patients with thyroid cancer. *Materials and methods:* We realized a retrospective study on patients with thyroid neoplasms who underwent surgery at the “Pius Brinzeu” County Clinical Emergency Hospital in Timisoara, Romania. A comparative analysis of some parameters between two groups, young patients (<45 years) versus patients ≥45 years, was performed. *Results:* A total of 211 patients met the study inclusion criteria, mostly females (86.26%) with a female/male ratio of 6.81:1. In patients <45 years old (25.64%), papillary thyroid carcinoma was identified in 51.85% of cases; in 53.85% of cases, the tumor was >1 cm; 13.46% had extrathyroidal extension (*p* = 0.0430); 21.15% capsule invasion (*p* = 0.1756); 23.08% lympho-vascular invasion (*p* = 0.0048); and 13.46% of cases locoregional nodal invasion (*p* = 0.0092). *Conclusions:* Thyroid cancer in young people was associated with chronic lymphocytic thyroiditis and tumor progression parameters, identifying more cases of extrathyroidal extension, locoregional nodal invasion, lympho-vascular invasion and perineural invasion in young patients compared to older ones. For a better understanding of this pathology and to improve diagnosis and therapeutic management, more studies are needed for these patients.

## 1. Introduction

Globally, in 2022, thyroid cancer accounted for 4.1% of human neoplasms, and the mortality rate was 0.5% [1]. During the last few years, the incidence of thyroid neoplasms has increased significantly, especially in young people [2], with 13% of tumors diagnosed in patients in their first decades of life being malignant thyroid tumors [3]. This increase in the incidence was mainly due to the improvement of detection methods by ultrasonography and other diagnostic techniques [4]. Although most patients with thyroid cancer have a favorable prognosis, and mortality remains relatively unchanged, it can strongly affect the quality of their lives [3,4]. In individuals aged 50 and above, thyroid neoplasms are often detected as an incidental finding, with treatment potentially posing greater risks than benefits [4]. However, the younger patients, often affected by more aggressive types of thyroid cancer, require better diagnosis and treatment options without any delay [3,4].

Thyroid neoplasms represent a heterogeneous group of tumors, with the most frequently encountered tumor subtype being papillary carcinoma (PTC), a well-differentiated tumor with a generally favorable prognosis. However, worse forms of thyroid cancer exist, such as anaplastic carcinoma (ATC); despite the low incidence, its mortality rate is very high. Moreover, even inside the same tumor, there can be heterogeneity, or more histological variants can be present simultaneously in the same patient, making therapeutic management very difficult [2]. Identifying the histopathological type of thyroid cancer guides the treatment and is relevant in assessing the prognosis for patients with thyroid tumors [3,4,5]. 

This paper aims to outline the social–demographical and clinical–pathological profiles of young patients with thyroid cancer and to assess several parameters in young patients, as compared with older patients.

## 2. Materials and Methods

### 2.1. Study Design and Participants

To achieve the proposed objectives, we conducted a retrospective study involving a cohort of patients diagnosed with thyroid cancer who underwent surgical resection and pathological examination at the “Pius Brinzeu” Emergency County Clinical Hospital Timisoara (PBECCHT). This study was carried out according to the principles of the Helsinki Declaration of good medical clinical practice, and all participants signed informed consent for the use of their medical data for scientific purposes at the time of admission.

Data were retrieved from the Pathological Anatomy Service database of PBECCHT. We performed a computerized search of pathology reports for the word “thyroid” between 1 January 2019 and 31 December 2020. We established the following *inclusion criteria*:

- Consecutive cases of malignant thyroid tumors: papillary carcinomas (PTCs), follicular carcinomas (FTCs), poorly differentiated carcinomas (PDTCs), anaplastic carcinomas (ATCs), medullary carcinomas (MTCs) and lymphomas; in addition, cases of well-differentiated thyroid tumors with uncertain malignant potential (UMP), confirmed by histopathological examination, were also included in the study.

The exclusion criteria were as follows:

- Nonneoplastic, inflammatory thyroid conditions;
- Benign thyroid tumors.

### 2.2. Data Sources and Variables of Interest

All data were obtained by analyzing the medical records of the patients, the accompanying sheets of the biopsy material and the pathological reports. Pathological diagnosis was established after standard histopathological processing of the surgical resection specimens/tissue fragments: 10% buffered formalin fixation, followed by paraffin embedding. From paraffin blocks, 4 μm thick sections were stained with the usual hematoxylin–eosin stain (HE). For difficult cases, new sections were cut from the paraffin blocks at 4 μm thickness, and additional immunohistochemical (IHC) stains were performed: TTF1, HBME-1, CD56, CK, Ki 67, p53, LCA, p63 or calcitonin. Immunoreactions were visualized with the polymer system, using DAB (diaminobenzidine) chromogen and hematoxylin counterstain, according to the manufacturer’s protocol.

### 2.3. Compiling the Database

The following sociodemographic parameters were recorded for each patient: age and sex of the patients; living environment; and clinical–morphological parameters, i.e., type of resected specimen (total/partial thyroidectomies, right/left lobectomy, excision biopsy), tumor size, histological subtype of tumors (according to the World Health Organization (WHO)’s *Classification of Tumours of Endocrine Organs*) [6], local extension of the tumor at the time of diagnosis (pT), capsular invasion, perineural invasion (Pn) and lympho-vascular invasion (L-VI), status of regional lymph nodes (pN) and associated pathology, as well as the IHC markers used. The staging of thyroid neoplasms was performed according to the TNM (*Tumor, Node, and Metastasis*) system developed by the AJCC (American Joint Committee on Cancer) [7], as follows:

- Primary tumor (pT):
  − pTX—primary tumor cannot be assessed;
  − pT0—no evidence of primary tumor;
  − pTI—tumor ≤2 cm in greatest dimension, limited to thyroid gland (pT1a—tumor ≤1 cm, pTIb—tumor >1 cm but ≤2 cm);
  − pT2—tumor >2 cm but ≤4 cm limited to the thyroid;
  − pT3: pT3a—tumor >4 cm limited to the thyroid, or pT3b—tumor of any size with gross extrathyroidal extension invading only strap muscles (sternohyoid, sternothyroid. thyrohyoid or omohyoid muscles);
  − pT4: pT4a—tumor of any size with gross extrathyroidal extension invading subcutaneous soft tissues, larynx, trachea, esophagus, or recurrent laryngeal nerve, or pT4b—tumor of any size with gross extrathyroidal extension invading prevertebral fascia or enclosing the carotid or mediastinal artery vessels.
- Regional Lymph Node (pN):
  − pNX—regional lymph nodes cannot be assessed;
  − pN0—no evidence of locoregional lymph node metastasis;
  − pN1: pN1a—metastasis to pretracheal, paratracheal, or prelaryngeal, or upper mediastinal lymph nodes; or pN1b—metastasis to unilateral, bilateral, or contralateral lateral neck lymph nodes or retropharyngeal lymph nodes.

For the comparative analysis of the parameters in young patients versus elderly patients, we used the age of 45 as a limit, and the study group was divided into two groups, depending on this threshold. We chose this cutoff of 45 years because it has been included in the thyroid cancer staging system since 1983, starting with the second edition of the AJCC Cancer Staging Manual [7], and the age of 45 is the age average of patients in most studies.

### 2.4. Statistical Analysis

The statistical analysis was performed using Microsoft Office Excel 2010 (Microsoft Corp., Redmond, WA, USA) and GraphPad Prism software, v8.2 (GraphPad Software Inc., San Diego, CA, USA). For comparing the results between the two groups, the Fisher test, Chi-square test and Student’s *t*-test were used. The results were considered statistically significant at values of *p* < 0.05.

## 3. Results

A total of 211 patients met the criteria for inclusion in the study. The demographic, clinical and pathological data of the patients included in the two groups, analyzed comparatively, are presented in Table 1.

### 3.1. Patients’ Age and Sex

**Figure 1:**
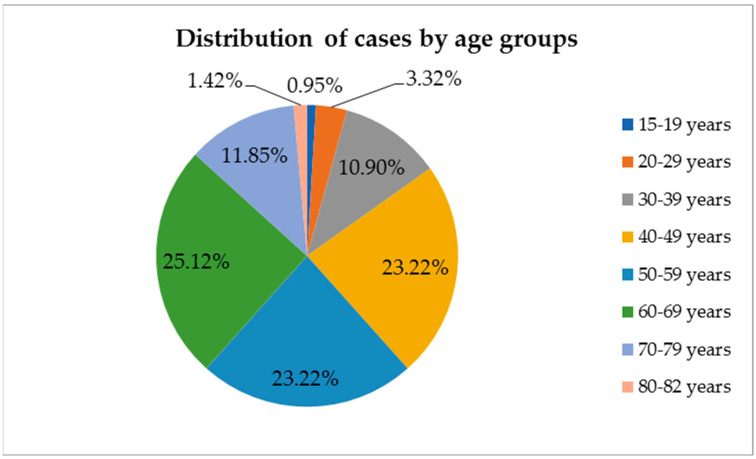
Distribution of thyroid tumors cases by age groups (number of cases = 211).

Of the total number of cases (211 patients with thyroid tumors) included in the study, 29 were males (13.74%) and 182 were females (86.26%), with an average age at the time of diagnosis of 53.91 years (between 15 and 82 years old). Most cases were identified in the 60–69 age group (53 cases; 25.12%) (Figure 1). In total, 52 patients (25.64%) were young adults aged <45 years, and 159 (75.36%) were adults aged ≥45 years, with the difference being statistically significant (*p* < 0.0001).

Most thyroid tumors were diagnosed in females, representing 80.77% (42/52 cases) of the first group and 88.05% (140/159) of the second group, without statistically significant differences between the two groups (*p* = 0.244). The female/male ratio was 6.81:1 for the entire lot of patients, 5.5:1 for group I and 7.3:1 for group II.

### 3.2. Living Environment of Patients

Among the patients aged <45 years, 56.86% (29/51) came from the urban environment; 43.14% (22/51) from rural areas; and in 1 case, the place of origin was not specified. From the group of patients aged ≥45 years, 64.33% (101/157) were from urban areas; 35.67% (56/157) were from rural places; and for 2 patients, the living environment was not specified (*p* = 0.4055). At the moment of diagnosis, the average age of patients with thyroid carcinoma was 51.35 years old for those from rural areas versus 55.44 years old for patients who came from an urban environment.

### 3.3. Method of Diagnosis and Association with Other Pathologies

A majority of cases (186/211, 88.15%) were diagnosed from total thyroidectomy specimens. In 11 cases (5.21%), a right lobectomy was performed, while in 12 cases (5.69%), a left lobectomy was conducted. Additionally, an excisional biopsy was performed in two cases (0.95%). In patients under 45 years old, malignant tumors were diagnosed on total thyroidectomy specimens in 40 cases (76.92%) and on lobectomy specimens in 12 cases (23.08%). In contrast, patients aged 45 years and older underwent a total thyroidectomy in 146 cases (91.82%), lobectomy in 11 cases (6.92%), and tumor fragment sampling in 2 cases (1.26%). The differences between the two age groups were statistically significant (*p* = 0.0058).

**Figure 2:**
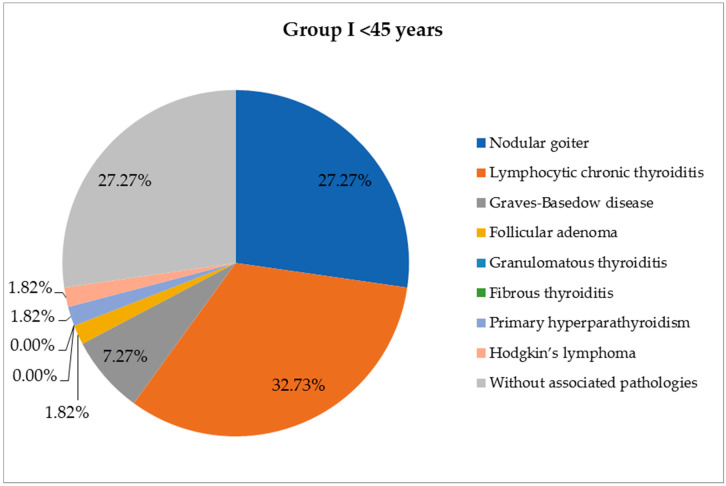
Association of thyroid cancer with other thyroid lesions in the age group <45 years (number of cases = 52).

**Figure 3:**
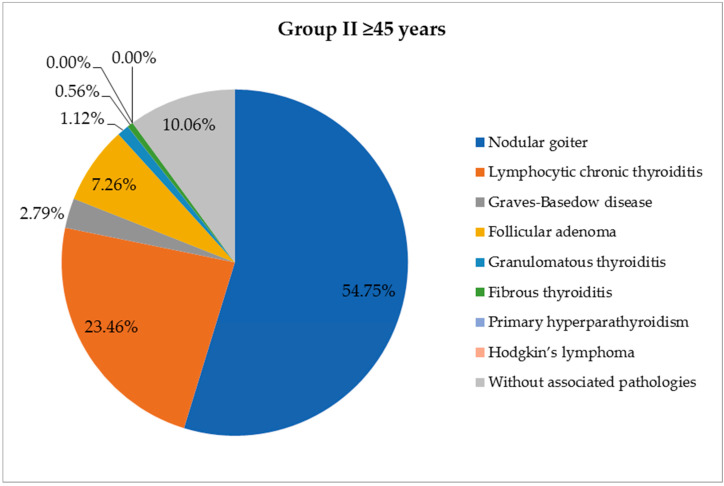
Association of thyroid cancer with other thyroid lesions in the age group ≥45 years (number of cases = 159).

A large part of the patients included in the study presented other thyroid lesions associated with the cancer (Table 1). In young patients, the most common pathology associated with the malignant tumor was chronic lymphocytic thyroiditis (18 cases, 32.73%), followed by primary thyroid follicular nodular disease (FND) (15 cases, 27.27%) and Graves–Basedow disease (4 cases, 7.27%). In elderly patients, malignant tumors appeared more frequently on the background of FND (98 cases, 54.75%); other associated thyroid lesions were chronic lymphocytic thyroiditis (42 cases, 23.46%), follicular adenoma (13 cases, 7.27%) and Graves–Basedow disease (5 cases, 2.79%), with statistically significant differences (*p* = 0.0004) between the two groups (Figure 2 and Figure 3).

### 3.4. Histopathological Types

**Figure 4:**
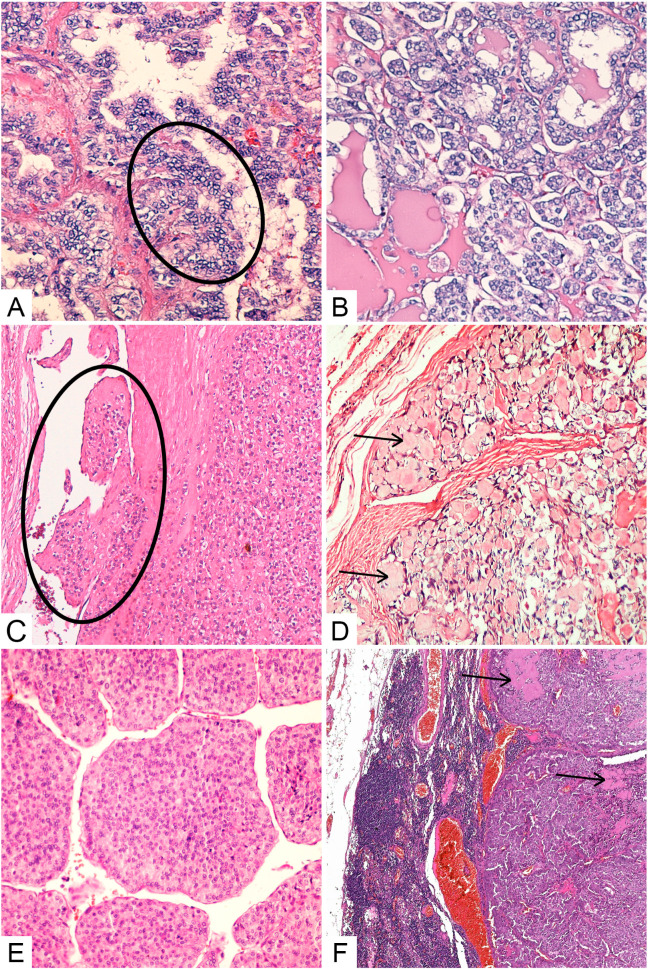
Histopathological types of thyroid cancer: (**A**) classic PTC subtype, showing papillary growth pattern lined by cells with characteristic nuclear features (circle), H&E staining, original magnification ×200; (**B**) FVPTC, H&E staining, original magnification ×200; (**C**) encapsulated, angioinvasive (circle) FTC, H&E staining, ×100; (**D**) MTC, classic variant, with amyloid deposits in the stroma (arrows), H&E staining, original magnification ×200; (**E**) PDTC—“insular” type, H&E staining, original magnification ×200; (**F**) lymph nodal metastasis of a MTC with amyloid deposits in the stroma (arrows), H&E staining, original magnification ×40.

**Figure 5:**
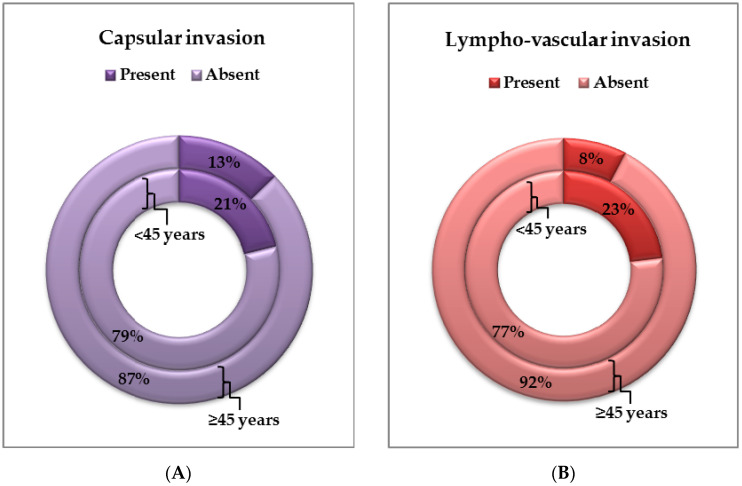
(**A**) Evaluation of capsule invasion in the two groups of patients. (**B**) Evaluation of lympho-vascular invasion in the two groups of patients.

In patients <45 years old, the most common histological type was papillary carcinoma (PTC) diagnosed in 28 cases (51.85%)—Figure 4A and Figure 5B; followed by papillary microcarcinoma (18 cases; 33.33%), well-differentiated tumor of uncertain malignant potential (WDT-UMP) in 5 cases (9.26%) and follicular carcinoma (FTC) identified in 3 cases (5.56%)—Figure 4C. In patients ≥45 years old, the following were identified: papillary microcarcinoma in 87 cases (50.88%); PTC in 56 cases (32.75%); UMP in 11 cases (6.43%), FTC in 8 cases (4.68%); medullary carcinoma (MTC) in 3 cases (1.75%, Figure 4D); anaplastic carcinoma (ATC), with 1 case of poorly differentiated carcinoma (PDTC), “insular” type, in 5 cases (2.92%, Figure 4E); and 1 case (0.58%) of thyroid follicular lymphoma, without statistically significant differences regarding the distribution of histological types of tumors between the two age groups (*p* = 0.1211). Histologically different thyroid tumors were diagnosed at the same time in 3 cases from group I and in 12 cases from group II. Papillary microcarcinomas were most frequently observed together with other histological variants, such as PTC, UMP, MTC or ATC. The microscopic images were obtained from the slides corresponding to the cases analyzed in this study.

PTC, papillary thyroid carcinoma; FVPTC, follicular variant of papillary thyroid carcinoma; FTC, follicular carcinoma; MTC, medullary thyroid carcinoma; PDTC, poorly differentiated carcinoma.

### 3.5. Tumor Extension, Lympho-Vascular Invasion and Perineural Tumor Invasion

Most tumors (70%) involved a single thyroid lobe (RTL). A total of 7/52 cases (13.46%) showed microscopic extrathyroidal tumor extension in patients <45 years and 6/159 cases (3.82%) in patients ≥45 years of age, with statistically significant differences (*p* = 0.0430).

Capsular involvement was identified in 11/52 (21.15%) cases from the first group and 20/157 (12.74%) cases from the second group (*p* = 0.1756), and lympho-vascular invasion was identified in 12/52 (23.08%) cases from the first group and 12/157 (7.64%) from the second group, respectively, with statistically significant differences (*p* = 0.0048) (Figure 4C and Figure 5A,B). In two cases of ATC diagnosed upon excisional biopsy, tumor extension at the time of diagnosis and capsular and lympho-vascular invasion could not be specified.

After analyzing the relationship between L-VI and tumor extension at diagnosis, capsular invasion and L-VI were correlated in both groups (*p* < 0.0001), 75% (9/12) of cases with L-VI presented capsule invasion in group I, and 67% (8/12) of cases in group II (Table 2). Also, L-VI and extrathyroidal tumor extension were correlated, with L-VI being absent in 97% (39/40) of the cases that did not present extrathyroidal tumor extension in group I (*p* < 0.0003), and in 99% (144/145) from cases in group II (*p* < 0.0001) (Table 3).

Perineural tumor invasion (Pn) was reported in 7 cases: 1/52 (1.92%) from group I and 6/157 (3.82%) cases from group II. We observed that, in the presence of Pn, capsular invasion (*p* = 0.0009) and L-VI (*p* = 0.0038) were more frequently encountered, with statistically significant differences. We also found an association between tumor extension and Pn (*p* = 0.0368) (Table 4).

### 3.6. TNM Staging

Regarding the pT parameter, 104 patients presented pT1a tumors: 17 (32.69%) from group I and 87 (54.72%) from group II. In 46 cases—15 (28.85%) from group I and 31 (19.15%) from group II—the tumors were classified as pT1b. Stage pT2 was assigned to 19 tumors: 5 (9.62%) from group I and 14 (8.81%) from group II. In 12 cases—5 (9.62%) from group I and 7 (4.40%) from group II—the tumors were staged as pT3a. A total of 8 cases—3 (5.77%) from group I and 5 (3.14%) from group II—presented tumors pT3b. Only one case in group II (0.63%) was assigned to stage pT4a, and none was assigned to stage pT4b. The comparative analysis of the two studied groups did not reveal any correlation (*p* = 0.1615). In 21 (9.95%) cases, the pT parameter could not be evaluated.

Our assessment of the pN parameter showed that 12 patients—7 (13.46%) from the first group and 5 (3.14%) in the second group—presented lymph node metastases (pN1). In group I, four cases were pN1a, and three cases were pN1b, as compared with group II, with three cases being pN1a and none being case pN1b. Regarding MTC, one case was classified as Nx, and the other two were staged as pN1. Lymph node involvement was more frequently reported in young patients (*p* = 0.0092).

Only one patient was known to have metastasis, located at the level of the T12 thoracic vertebra, thus belonging in the pM1 stage.

## 4. Discussion

According to the fifth edition of the *WHO Classification of Endocrine and Neuroendocrine Tumors*, thyroid cancer represents an entity that includes a wide range of histological aspects and molecular and genetic characteristics [6,8,9]. The global incidence of thyroid cancer is increasing [10,11], with the people most affected being females, with a female/male ratio of 2.97:1 [12], and with a higher rate of diagnosed tumors in developed countries [3,13,14]. Regarding tumor pathology in young patients, thyroid cancer ranks second after breast cancer [15], but it is the most common thyroid malignancy in the pediatric population [16,17].

### 4.1. Patients’ Age, Sex and Living Environment

For patients with thyroid cancer, the age at initial diagnosis is considered a prognostic factor in TNM staging [18], with mortality being progressively higher with advancing age, especially after the age of 35–40 years [7]. Thyroid cancer can occur at any age, but the average age at diagnosis is 50 years old, and the highest frequency is observed between 45 and 54 years of age [5]. According to Kitahara et al., the incidence of thyroid cancer peaks around the age of 55 years in females and 65 in men, and then it decreases with advancing age [19]. In our study, the average age at diagnosis was 54 years old, and the maximum incidence was found in the 60–69 age group; these results are similar to the results from Nguyen et al.’s study, which recorded an average age of 50 years old at diagnosis [5]. In several studies, thyroid cancer has been observed to have a higher incidence in patients aged ≥45 years [20,21]. Similar to the literature data, in our study, thyroid cancer cases were more frequent in patients aged ≥45 years (75.36%, *p*< 0.0001). In this study, we chose the age limit of 45 years for the following reasons: the purpose of the study was to outline the profile of young patients (under 45 years), the age of 45 years is the average age of patients in several studies and because this cutoff of 45 years was considered a prognostic factor starting with the second edition of the AJCC Cancer Staging Manual [7]. Moreover, the age <45 years was related to the increased risk of lymph node metastasis in patients with papillary thyroid carcinoma [22,23]. Establishing the threshold limit for the age at diagnosis has been the subject of research in numerous studies. In most staging systems, age 45 is used to differentiate low-risk thyroid cancer from high-risk thyroid cancer [7,20]. However, recent studies have shown that age is insufficient in predicting the prognosis for patients with thyroid cancer [18]. There is also an argument that a binary variable for age may not be appropriate and that a staging system using age as a continuous variable could provide more prognostic information [18,24]. The mortality due to the thyroid cancer increases progressively with age, but no single age cutoff has been reported that accurately stratifies patients into risk categories [7]. Recent studies have demonstrated that the use of the age threshold of 55 years would have a more important prognostic value [24,25,26,27]; therefore, the latest (i.e., eighth) edition of TNM introduced a change in age cutoff from 45 to 55 [7,28]. Nevertheless, current studies highlight that, due to the down-staging of patients with thyroid cancer when performing the TNM 8th edition classification, patients predisposed to recurrence may be overlooked, affecting therapeutic decisions at the time of disease diagnosis [29].

According to Nguyen et al., thyroid cancer is more common among females, with a female/male ratio of 3:1 [5]. In our study, the female/male ratio was 6.81:1, which is significantly higher compared to the ratio mentioned in the previous study. In females, the tumor was diagnosed more frequently between the ages of 15 and 49, while in males, between the ages of 50 and 69.

Regarding the patients’ environment, we found a higher incidence of thyroid neoplasms in the urban areas (about 40%). This result is comparable to that of Guay et al.’s result, which indicates at least a 25% higher incidence of these tumors in urban patients [21]. In our study, we observed differences in the average age at diagnosis. Specifically, patients from rural areas tend to develop thyroid carcinoma at a younger age on average, in contrast to patients from urban environments.

### 4.2. Associated Thyroid Pathology and Type of Surgical Intervention

Concerning the pathology associated with thyroid cancer, the incidence of malignancy in patients with thyroid follicular nodular disease (FND) is appreciable. According to some authors, the incidence of thyroid carcinoma associated with FND is between 8.6 and 13% [30]. Graves–Basedow disease has been associated with an increased risk of malignancy. Some authors report a more aggressive evolution of thyroid cancer in patients with hyperthyroidism [31]. Autoimmune/Hashimoto thyroiditis is considered to be a risk factor for the development of thyroid lymphomas (reported in 80% of cases) [32], but it was also diagnosed in 27.6% of cases with PTC [33]. In our study, we observed that FND, chronic lymphocytic thyroiditis, Graves–Basedow disease and follicular adenoma were the most frequently associated lesions. Thyroid neoplasms were more often associated in group I with lesions of lymphocytic thyroiditis and in group II with FND; Graves-Basedow disease was identified more frequently in group I, and follicular adenoma in group II, with the difference being statistically significant (*p* = 0.0004).

The identification of the tumor subtype has therapeutic implications. In PTC, the most common treatment is surgery—total thyroidectomy/lobectomy. Total thyroidectomy offers advantages such as the use of radioactive iodine as adjuvant therapy and allows for accurate postoperative surveillance of thyroglobulin levels; on the other hand, lobectomy decreases the risk of permanent hypoparathyroidism and recurrent lesions of the laryngeal nerve [34]. However, it is not clear whether total thyroidectomy provides better overall survival or recurrence-free survival than lobectomy [20,35]. In our study, total thyroidectomies were performed in 88.15% of cases, in 23.08% of the patients from the first study group and in 6.92% of those in the second group, with the differences being statistically significant (*p* = 0.0058).

### 4.3. The Histological Subtype of Thyroid Cancer

PTC is the most common form of thyroid cancer, accounting for 80–85% of all cases, and has the best prognosis, especially in patients under 45 years of age [34]. Regarding the histological type, in our study, PTC was diagnosed in 86.30% of cases. Histopathological PTC presents a complex morphological aspect, with a series of variants being described as follows: classic variant, encapsulated, follicular variant, clear cell variant, microcarcinoma (diameter ≤ 1 cm), etc. Among the forms classified as aggressive are the variants with tall cells, columnar cells, “hobnail”, solid or diffuse, and sclerosing [9,17]. Aggressive forms have important clinical significance, poor prognosis and intermediate risk of recurrence, and their incidence appears to be increasing [9]. Moreover, within the same tumor, several variants can coexist; for example, the classic one is often accompanied by the follicular one. The simultaneous presence of two different histological variants may be important if one of them is more aggressive [2].

In total, 5.02% of our cases were represented by FTC. In the literature, FTC accounts for approximately 5–10% of thyroid malignancies in non-endemic goiter areas of the world [36]. This is the second most common histological form of differentiated thyroid cancer, with a worse prognosis compared with the PTC. Although the incidence of thyroid cancer has nearly tripled in the last decades, the incidence of FTC has remained relatively stable, even decreasing due to iodine supplementation in the general population’s diet [37]. In addition, changes in diagnostic criteria over time have made it difficult to analyze the epidemiology of this form of cancer, given that some FTCs have been reclassified as papillary carcinomas, the follicular variant (FVPTC) [38]. The histopathological examination is an extremely important tool for establishing the diagnosis of thyroid cancer. The histopathological subtype of FTC cannot be diagnosed exclusively on cytological aspects. The identification of certain aspects, such as vascular and capsular invasion, is necessary for establishing a diagnosis of a malignant tumor [37]. According to the WHO, capsular invasion is defined as the complete crossing of the capsule by a part of the tumor, while vascular invasion is represented by the presence of malignant cells adhering to the vascular walls [38]. Thus, an examination of multiple sections of the lesion and the entire tumor capsule is necessary for diagnosis [38].

Anaplastic thyroid carcinomas (ATCs) are a rare class of malignant tumors, representing only 1–2% of thyroid carcinomas, but being responsible for 14–39% of the deaths caused by them [39]. The average survival after diagnosis is 5–6 months, and 2-year survival can be found in only 10–15% of cases [39]. Fortunately, the incidence is decreasing, especially due to the early detection of small tumors by ultrasonography, as well as the reduction in the incidence of endemic goiter by iodine supplementation (goiter being correlated with the occurrence of ATC) [39]. In our study, in patients <45 years old, we did not identify any case of thyroid tumor with a low degree of differentiation, and in patients ≥ 45 years old, we identified only five cases (2.36%), with four being ATC and one with features of PDTC. Our results are comparable with data from the literature showing that a small percentage of 2–15% of thyroid tumor cases are described as PDTC [40].

Medullary thyroid carcinoma (MTC) is a rare type of cancer, representing 2–4% of thyroid carcinomas, originating from parafollicular C cells [41]. In our study, similar to data from the literature, this type of tumor was diagnosed in 1.42% of cases. Histologically, this carcinoma is usually infiltrative, poorly delimited, and made up of cells arranged in nests (similar to some neuroendocrine tumors), within a fibrous stroma, sometimes containing amyloid (a pathognomonic aspect, with amyloid being a pre-procalcitonin deposit). However, sometimes, they show uncharacteristic histological features, such as the formation of pseudo papillae (mimicking PTC), or having follicular architecture. In these cases, IHC can make a difference; these cells are immunohistochemically positive for calcitonin, chromogranin A and carcinoembryonic antigen (CEA), markers which are also useful for postoperative monitoring [42].

### 4.4. Lympho-Vascular Invasion, Extrathyroidal Extension and Lymph Node Metastases

In our study, 23.08% of patients <45 years old presented with L-VI. Lympho-vascular invasion was strongly correlated with capsule infiltration (*p* < 0.0001), extrathyroidal extension (*p* < 0.0001) and Pn (*p* = 0.0038). Lympho-vascular invasion, including only lymphatic invasion, is associated with a risk of persistent/recurrent disease in PTC patients; thus, it could be included in risk stratification systems and could guide decision-making for these patients [43]. Moreover, we found a more pronounced tendency toward extrathyroidal extension among patients with perineural invasion, with the results being statistically significant (*p* = 0.0368). Other studies report correlations between L-VI and some clinical and pathological features, such as extrathyroidal extension, Pn and capsular infiltration [44]. According to our results, more cases of the extrathyroidal extension were identified in patients under 45 years of age. Thus, 13.46% of cases with extrathyroidal extension were recorded in group I, as compared to 3.82% in group II, with the results being statistically significant (*p* = 0.0430). Moreover, locoregional nodal invasion (pN1 stage) was more frequent in group I: among the cases in which nodule dissection was performed, 13.46% presented locoregional lymph node invasion, as compared to 3.14% in group II, the differences being statistically significant (*p* = 0.0092). The results of the study conducted by Bestepe et al. show that there is a tendency for extrathyroidal extension and nodal metastases to occur more frequently in patients under 39 years of age, but without reporting a statistically significant relevance [45]. In another study, Kaouache et al. concluded that there is a higher frequency of capsular invasion and lympho-nodal invasion in patients younger than 40 years of age [46]. According to Wang et al. lympho-vascular invasion, absences of tumor capsule and extrathyroidal extension have been reported as predictors of nodal disease [47]. However, the prognostic significance of regional lymph node metastases in differentiated thyroid cancer is still controversial (47). It is known that PTC patients with clinically apparent lateral lymph node metastases (pN1b) have an unfavorable prognosis [47,48]. In our study, all pN1b cases were diagnosed only in young patients. Some authors concluded that the aggressiveness of N1b carcinoma is also varied, depending on differences in the size, number and invasiveness of metastases [47,48].

*Study limitation:* Our study had several limitations, such as the absence of data on the treatment received by the patients or their evolution after surgery that would make it possible to correlate the obtained results with survival and would significantly improve our work. Unfortunately, at present, in Romania, we do not have the possibility to follow the evolution of patients, because patients from all over the country can present themselves at regional hospitals, and the lack of an up-to-date National Cancer Registry, which would make it possible to complete and uniformly report cases and of cancer types, does not allow for patient follow-up or for an accurate assessment of disease incidence, prevalence and mortality. Furthermore, our study is limited by the small number of patients diagnosed in a single hospital and the fact that the analysis was performed retrospectively.

## 5. Conclusions

In this study, thyroid cancer cases were diagnosed more frequently in patients aged 45 years and older. In young patients, thyroid cancer has been associated with lesions such as chronic lymphocytic thyroiditis and parameters indicative of aspects of tumor progression, such as extrathyroidal extension, locoregional lymph node invasion, lympho-vascular invasion and perineural invasion. Our findings highlight the need to monitor the incidence of thyroid cancer in young adults to assess whether changes in screening practices are needed and to raise awareness among physicians of the increasing incidence of thyroid cancer in this patient group. Therefore, our study should be complemented by additional larger studies that outline the clinical–pathological profile of patients with thyroid neoplasms.

## Figures and Tables

**Table 1:** Comparative analysis of the two study groups using the Chi-square test and the Fisher exact test (number of cases = 211).

	Parameters	Group I <45 Years (%)	Group II ≥45 Years (%)	*p*-Value
**Sex**	Men	10 (19.23)	19 (11.95)	0.2444
	Females	42 (80.77)	140 (88.05)	
**Origin**	Urban	29 (56.86)	101 (64.33)	0.4055
	Rural	22 (43.14)	56 (35.67)	
**Type of intervention**	Total thyroidectomy	40 (76.92)	146 (91.82)	0.0058
	Subtotal right lobectomy	7 (13.46)	4 (2.52)	
	Subtotal left lobectomy	5 (9.62)	7 (4.40)	
	Tumor fragments (excision biopsy)	0	2 (1.26)	
**Associated thyroid pathology**	Thyroid follicular nodular disease (FND)	15 (27.27)	98 (54.75)	0.0004
	Lymphocytic chronic thyroiditis	18 (32.73)	42 (23.46)	
	Graves–Basedow disease	4 (7.27)	5 (2.79)	
	Follicular adenoma	1 (1.82)	13 (7.26)	
	Granulomatous thyroiditis	0	2 (1.12)	
	Fibrous thyroiditis	0	1 (0.56)	
	Primary hyperparathyroidism	1 (1.82)0	0	
	Hodgkin’s lymphoma	1 (1.82)	0	
	Without relevant associated pathologies	15 (27.27)	18 (10.06)	
**Histological subtype of thyroid cancer**	Papillary microcarcinoma	18 (33.33)	87 (50.88)	0.1211
	Papillary carcinoma (PTC)	28 (51.85)	56 (32.75)	
	Follicular carcinoma (FTC)	3 (5.56)	8 (4.68)	
	Well-differentiated tumor of uncertain malignant potential (WDT-UMP)	5 (9.26)	11 (6.43)	
	Poorly differentiated (PDTC) and anaplastic carcinoma (ATC)	0	5 (2.92)	
	Medullary carcinoma (MTC)	0	3 (1.75)	
	Thyroid lymphoma	0	1 (0.58)	
**Location of the tumor**	One lobe	34 (65.38)	111 (70.70)	0.0430
	Both lobes	11 (21.15)	40 (25.48)	
	Extrathyroidal	7 (13.46)	6 (3.82)	
**Capsular invasion**	Present	11 (21.15)	20 (12.74)	0.1756
	Absent	41 (78.85)	137 (87.26)	
**Lympho-vascular invasion**	Present	12 (23.08)	12 (7.64)	0.0048
	Absent	40 (76.92)	145 (92.36)	
**Perineural tumor invasion**	Present	1 (1.92)	6 (3.82)	0.6835
	Absent	51 (98.08)	151 (96.18)	
**pT classification**	pTx	7 (13.46)	14 (8.81)	0.1615
	pT1a	17 (32.69)	87 (54.72)	
	pT1b	15 (28.85)	31 (19.50)	
	pT2	5 (9.62)	14 (8.81)	
	pT3a	5 (9.62)	7 (4.40)	
	pT3b	3 (5.77)	5 (3.14)	
	pT4a	0	1 (0.63)	
**Regional lymph nodes status (pN)**	pNx	36 (69.23)	135 (84.91)	0.0092
	pN0	9 (17.31)	19 (11.95)	
	pN1	7 (13.46)	5 (3.14)	

PTC, papillary thyroid carcinoma; FTC, follicular thyroid carcinoma; WDT-UMP, well-differentiated tumor of uncertain malignant potential; PDTC, poorly differentiated thyroid carcinoma; ATC, anaplastic thyroid carcinoma; MTC, medullary thyroid carcinoma; pT, tumor extension; pN, lymph nodes status.

**Table 2:** The relationship between capsule invasion and lympho-vascular invasion in the two groups of patients.

Group I <45 Years Old				Group II ≥45 Years Old			
	L-VI Present	L-VI Absent	*p*-Value		L-VI Present	L-VI Absent	*p*-Value
Capsular invasion present	9	2	<0.0001	Capsular invasion present	8	12	<0.0001
Capsular invasion absent	3	38		Capsular invasion absent	4	133	

L-VI: lympho-vascular invasion.

**Table 3:** The relationship between extrathyroidal tumor extension and lympho-vascular invasion in the two groups of patients.

Group I <45 Years Old				Group II ≥45 Years Old			
	L-VI Present	L-VI Absent	*p*-Value		L-VI Present	L-VI Absent	*p*-Value
Extrathyroidal tumor extension present	6	1	0.0003	Extrathyroidal tumor extension present	5	1	<0.0001
Extrathyroidal tumor extension absent	6	39		Extrathyroidal tumor extension absent	7	144	

L-VI: lympho-vascular invasion.

**Table 4:** The relationship between perineural invasion (Pn) and other parameters analyzed for the entire group of patients.

	Pn Present	Pn Absent	*p*-Value
**Capsular invasion—present**	5	26	0.0009
**Capsular invasion—absent**	2	176	
**L-VI—present**	4	20	0.0038
**L-VI—absent**	3	182	
**Extrathyroidal extension**	2	11	0.0368
**Involvement of both thyroid lobes**	2	49	
**Involvement of a single thyroid lobe**	3	142	

L-VI, lympho-vascular invasion; Pn, perineural invasion.

## Data Availability

All data generated or analyzed during this study are included in this published article and can be provided if needed or requested by the reviewer.
